# Consumers lived experiences and satisfaction with sub-acute mental health residential services

**DOI:** 10.1007/s00127-024-02631-3

**Published:** 2024-03-08

**Authors:** S. Waks, E. Morrisroe, J. Reece, E. Fossey, L. Brophy, J. Fletcher

**Affiliations:** 1 Discipline of Clinical Psychology, Graduate Schools of Health, University of Technology Sydney, Chippendale, NSW Australia; 2 Centre for Mental Health, Melbourne School of Population and Global Health, The University of Melbourne, Melbourne, VIC Australia; 3 Discipline of Psychological Science, Australian College of Applied Professions, Melbourne, VIC Australia; 4 Department of Occupational Therapy, School of Primary and Allied Health Care, Monash University, Frankston, VIC Australia; 5 The ALIVE National Centre for Mental Health Research Translation, The University of Melbourne, Melbourne, VIC Australia; 6 Social Work & Social Policy, School of Allied Health, Human Services and Sport, La Trobe University, Melbourne, VIC 3086 Australia

**Keywords:** Consumer experiences, Mental illness, Recovery-oriented practice, Sub-acute residential mental health services, Evaluation

## Abstract

**Purpose:**

Sub-acute recovery-oriented facilities offer short-term residential support for people living with mental illness. They are generally highly regarded by consumers, with emerging evidence indicating that these services may support recovery. The aim of the current study was to explore the relationship between personal recovery and consumers’ satisfaction with sub-acute residential services, and consumers’ views about service features that aid recovery.

**Methods:**

Consumers at 19 adult Prevention and Recovery Care Services in Victoria, Australia, were invited to complete measures containing sociodemographic information and measures on personal recovery and wellbeing. After going home, participants were invited to complete measures on service satisfaction and experience.

**Results:**

Total and intrapersonal scores on the personal recovery measure increased significantly between Time 1 and Time 2, indicating marked improvement. Personal recovery and satisfaction measures were moderately to strongly correlated. Thematically analysed open-ended responses revealed themes of feeling connected, finding meaning and purpose, and self-empowerment as important aspects of these services, with some recommendations for improvements.

**Conclusion:**

Sub-acute residential mental health care may support individuals’ personal recovery; consumer satisfaction indicates these services also offer an acceptable and supportive environment for the provision of recovery-oriented care. Further exploring consumers’ experiences of sub-acute residential services is essential to understand their effectiveness, opportunities for improvement and intended impacts on personal recovery.

## Introduction

Recovery-oriented practice has become a core component of the mental health systems in many countries in Europe and North America, as well as in Australia [1–3]. Personal recovery focuses on the strengths of people with mental illness for building fulfilling lives not defined by their illness [4]. Recovery-oriented practice refers to person-centred practices through which practitioners employ skills, values, and behaviours that support individuals in their recovery, moving away from evaluating services and individuals’ recovery based on clinical outcomes, for example reduced admissions or clinical symptoms [5]. There is also a growing evidence base of the value to consumers of recovery-oriented practice; for example, studies report that positive modelling from peer workers increases consumers’ sense of hope, belonging, and quality of life [6, 7]. Improved personal recovery outcomes have also been reported by consumers following training of staff in recovery-oriented practice [8].

In Australia, personal recovery and recovery-oriented practice have influenced mental health policy and service delivery [9, 10]. This includes the development of Prevention and Recovery Care (PARC) services in the state of Victoria. These sub-acute community based residential care services aim to support people with mental illness either as a ‘step-up’ from the community when they require extra support, potentially to avoid a hospital admission, or as a ‘step-down’ from an inpatient mental health ward to assist with transition back to community living. PARC services provide recovery-oriented care and operate under a partnership model between public clinical mental health services and community managed mental health support providers, with staff from both service types [11, 12]. With a most commonly 10-bed capacity, PARC services typically provide short-term support for 7–28 days. During their stay, consumers are encouraged to establish or maintain links with their families, friends, and other supports, and to participate in community life, including work or study [9].

The availability of PARC services for adults has been expanding in Victoria since 2003, yet the current evidence base for effectiveness of sub-acute services is limited [12]. A growing body of international evidence supports community-based residential services as an alternative to standard inpatient care but, whilst showing promise, there is significant encouragement in various reviews to support further research [13, 14]. The value of identifying consumer experiences of supported accommodation services to contribute to service improvement efforts has also been identified, particularly in how these services might further embed recovery oriented practice [15]. Some emerging evidence indicates that PARC services and similar services promote increased recovery for consumers [16–20]. Significant improvements in consumer self-reported psychological distress, self-efficacy, and work and social adjustment were also reported in Western Australia’s first sub-acute community residential service [21]. High levels of satisfaction with PARC-type services have been reported, with time and space to recuperate, gaining perspective, increased understanding and resilience, renewed focus and direction, and improved routines, confidence, and connections being the most valued outcomes [21, 22]. None of these studies used explicit measures of recovery, although their qualitative findings have indicated that consumers linked benefits to supporting their personal recovery.

To further build the evidence base for sub-acute community residential services, a state-wide evaluation of the appropriateness, effectiveness, and efficiency of PARC services in Victoria was undertaken in partnership among several Victorian universities, service provider organisations, and a research team that included lived experience researchers. Nineteen adult PARC services participated in this evaluation from 2016 to 2019, which consisted of a series of interrelated studies. Reporting to date includes a service mapping study of the structure, function, resourcing and quality of care provided by PARC services, which found that these services are operating according to the guidelines [11] and a PARC service typology based on significant differences in service characteristics such as the year in which the PARC service was opened, the living environment, proportion of admissions that were step-down from an inpatient unit, and the regularity with which families were invited to care meetings [12]. Outcomes of a matched pairs comparison between PARC and inpatient services found that in the year following a PARC service stay consumers are significantly less likely to have an inpatient admission and less likely to be on a Community Treatment Order (CTO) [23] and a study investigating similarities and differences between people accessing PARC services and inpatient units in Victoria found that they serve an overlapping group of consumers with some distinguishable differences regarding gender and diagnosis [14]. Lastly, a longitudinal study examining the quantitative recovery and wellbeing outcomes of PARC services for participants found that there were significant improvements for consumers relating to their personal recovery, quality of life, mental health and wellbeing [24]. None of the papers to date have reported consumer experiences of PARC services over the 12-month period.

Engagement of consumers in research to understand their experiences of services is crucial to bring about the attitudinal change and service delivery reform necessary for implementation of recovery-oriented practice. This paper aims to examine the relationship between personal recovery and consumer satisfaction with PARC services through exploring consumers’ views as measured by consumer self-reporting on recovery, service use, wellbeing, and quality of life.

## Materials and methods

### Study design

The study design was a mixed methods evaluation as part of a longitudinal cohort study. A detailed description of the study design, recruitment, and data collection can be found in Brophy, Fletcher [24]. The current paper focuses on data collected during participants’ first week in the PARC services (Time 1), then 1-week post-discharge (Time 2). Ethics approval was granted by Monash University Health Sciences Human Ethics Sub-Committee as part of the state-wide PARC Services Project Evaluation across Victoria (number: HREC/16/MonH/393) and governance approval was granted by the health services involved.

### Setting

At the commencement of this study in 2016, there were 19 sub-acute adult PARC services, offering a 28-day maximum stay. One of the participating PARC services was a woman-only service; the others were mixed gender environments. Three of these PARC services were located in inner-city areas, 12 in suburban areas, and the remaining four in regional areas of Victoria.

### Recruitment and data collection

All participants in this study were required to be 18 years of age or older, have sufficient English proficiency to participate, and have been at the PARC service for less than 7 days prior to being invited to participate. Recruitment and interviews were conducted by a team of researchers, some of whom were lived experience researchers and all were trained by lived experience researchers. Researchers held an introductory meeting at each of the 19 PARC services, and eligible consumers present were invited to participate. Researchers obtained written informed consent from each participant, facilitated their completion of a set of measures on an iPad, collected their contact information, and provided each participant with a $25 gift voucher for their time. One week following participants’ exit from the PARC service (following confirmation of the persons exit), a lived experience researcher began to contact participants via telephone, email, or post as per their preference to complete a second survey. They were able to complete the survey with support or independently. Participants received a $10 gift voucher for their time completing measures at Time 2. All data were securely stored and deidentified.

### Measures

Three of the outcome measures administered are the focus of this study: the Questionnaire about the Process of Recovery (QPR) [25], the INSPIRE [26], and the Mind Australia Satisfaction Survey (MASS) [27]. QPR data were collected at both Time 1 and Time 2 whilst the MASS and INSPIRE were completed at Time 2 only. In addition to these measures, sociodemographic questions were completed at Time 1. A full description of all measures used at all four timepoints in the parent longitudinal study can be found in Brophy, Fletcher [24].

The QPR is a 22-item measurement tool developed in collaboration with consumers. It is a widely used tool for assisting consumers to set and evaluate goals, for promoting recovery in service evaluation, and in research [25, 26]. The QPR has been validated as a measure of personal recovery in the UK and Australia [8, 26]. The QPR is comprised of two subscales measuring intrapersonal and interpersonal recovery processes, with each item rated on a 5-point Likert scale with higher scores indicating increased sense of personal recovery. QPR scores range from 0 to 88 for the total QPR score, 0–68 for the intrapersonal subscale, and 0–20 for the interpersonal subscale.

The INSPIRE explores a consumer’s experiences of the support received from a mental health worker in their recovery and was chosen for this study since PARC services aim to provide recover-oriented care. INSPIRE was developed as part of the REFOCUS programme with input from consumers, mental health professionals, and researchers and has been psychometrically evaluated [26]. INSPIRE is based on the five personal recovery processes previously identified in the CHIME Framework: Connectedness, Hope, Identity, Meaning, and Empowerment [28]. The 27-item version of INSPIRE is comprised of support and relationship subscales which measure the degree of support provided by a mental health worker and the relationship built with that worker. INSPIRE scores range from 0 to 100 for both subscales.

The MASS is a 12-item self-report questionnaire that has been used by Mind Australia (a partner agency in this study) to assess consumer satisfaction; it was adapted from a satisfaction survey developed by Rethink in the UK [27] and has subsequently been used in other studies to measure service delivery satisfaction [8, 29]. The MASS includes 9 closed-ended questions with responses on a five-point Likert scale, ranging from 1 Strongly Disagree to 5 Strongly Agree. The remaining three items invite open-ended responses: “What has been the most helpful thing about your experience with the service?”, “If you could change anything about the service what would it be?”, and “Any other comments and feedback?”.

### Data analysis

Initially, quantitative data were screened for assumptions underlying the chosen analyses, including assessment of outliers and missing values. Sociodemographic data were compared between participants who did and did not complete assessment at Time 2 to identify any systematic differences between completers and non-completers. A *t* test was used to examine differences between QPR scores of personal recovery change at both timepoints. Change scores for the QPR (total and subscales) were calculated by subtracting the score at Time 2 with the score at Time 1. Pearson’s correlation was used to calculate the associations between personal recovery change (QPR) and recovery (INSPIRE), and personal recovery change and service satisfaction (MASS). A single-factor between-subjects ANOVA was used to identify differences in the QPR, INSPIRE, and MASS scores amongst three service typologies previously reported [12]. These clusters are separated by the length of time the PARC service has been open, the number of step-down admissions, the quality of environment and the involvement of family in care meetings [12].

Qualitative data from the MASS were analysed thematically using a general inductive approach [30] by two team members: the first author (SW), a lived experience researcher who conducted the majority of interviews across timepoints with participants, and a qualitative researcher with mental health practitioner experience (EF). The data were initially coded inductively by the first author to identify themes from the responses to the open-ended questions included in the MASS, and then shared and discussed with the second researcher (EF). The themes were then further developed with reference to the CHIME framework, examining whether themes mapped onto the CHIME framework of personal recovery, if any new themes emerged and how consumer experiences of PARC services interconnected with processes thought to support personal recovery [28].

## Results

### Quantitative results

Table 1 presents the sociodemographic data for all participants at Time 1 and for participants who completed surveys at Time 2.

**Table 1:** Sociodemographic data of participants

	Time 1 (*n* = 298)		Time 2 (*n* = 186)	
	Freq	%	Freq	%
Gender				
Male	114	38.3	66	35.5
Female	170	57.0	111	59.7
Other	14	4.7	9	4.8
Age				
20–31 years	73	24.5	45	24.2
32–41 years	74	24.8	36	19.4
42–50 years	77	25.8	50	26.9
51–65 years	66	22.1	49	26.3
Missing	8	2.7	6	3.2
Country of birth				
Not Australia	45	15.1	28	15.1
Australia	246	82.6	152	81.7
Missing	7	2.3	6	3.2
Highest level of education				
High school or less	159	53.4	87	46.8
Diploma, certificate, or university	131	44.0	93	50.0
Missing	8	2.7	6	3.2
Marital status				
Single	170	57.0	112	60.2
Married or de facto	54	18.1	31	16.7
Separated, divorced, or widowed	66	22.1	37	19.9
Missing	8	2.7	6	3.2
Children				
Yes	150	50.3	91	48.9
No	141	47.3	89	47.8
Missing	7	2.3	6	3.2

A total of 298 participants provided some data at Time 1 with 186 participants providing data at Time 2. Data at both timepoints was provided by 181 participants. No missing data were estimated and all assumptions underlying the chosen analyses were met. No outliers justified deletion.

Analysis of participants who provided data at Time 2 in comparison with those who did not complete Time 2 (Table 1) found no significant associations on any demographical variables except education level. Participants who provided data at Time 2 were more likely to have a tertiary qualification (51% versus 35%), *χ*^2^ (1, *n* = 290) = 7.50, *p* = 0.006. No significant differences were found between those who provided data at Time 2 and those who did not on the three QPR sub-scales.

Table 2 presents means and standard deviations for all measures completed at Time 1 and Time 2 for participants who provided responses at both timepoints. Significant increases were seen between Time 1 and Time 2 for the total QPR score, *t*(180) = 4.84, *p* < 0.001, *d* = 0.36, 95% CI [0.21, 0.51], and for intrapersonal QPR scores, *t*(180) = 5.21, *p* < 0.001, *d* = 0.39, 95% CI [0.24, 0.54]. No significant difference was found between interpersonal QPR scores at Time 1 and at Time 2. The mean MASS rating of PARC services was 36.96, and the mean INSPIRE rating was 83% for the relationships subscale and 71% for Support sub-scale.

**Table 2:** Descriptive data for quantitative measures (*n* = 181)

Scale	Time 1			Time 2			Change score		
	*n*	*M*	SD	*n*	*M*	SD	*n*	*M*	SD
QPR total	181	72.48	15.5	181	78.2	16.02	181	5.72	15.9
QPR intrapersonal	181	57.53	14.21	181	63.04	13.98	181	5.51	14.23
QPR interpersonal	181	18.98	2.78	181	19.14	3.14	181	0.16	3.14
MASS				170	36.96	6.21			
INSPIRE relationships				167	83.27	19.07			
INSPIRE support				168	70.88	23.23			

Table 3 presents the associations between the QPR change scores, and the INSPIRE and MASS scores at Time 2. The total QPR change score, and the intrapersonal and interpersonal QPR change scores were all moderately to strongly correlated. All QPR change scores were weakly correlated with the MASS score and with both subscales of the INSPIRE. The MASS was moderately to strongly correlated with both subscales of the INSPIRE. Both INSPIRE subscales were also strongly correlated with each other. No significant differences were found in the QPR, INSPIRE, or MASS in the context of the PARC service clusters [12].

**Table 3:** Correlations among quantitative measures

	Change in total QPR score	Change in intrapersonal QPR score	Change in interpersonal QPR score	Sum of all MASS scores	INSPIRE Relationships subscale	INSPIRE Support Subscale
Change in total QPR score						
Change in intrapersonal QPR score	.99**					
Change in interpersonal QPR score	.68**	.57**				
Sum of all MASS scores	.39**	.38**	.35**			
INSPIRE relationships subscale	.37**	.37**	.26**	.74**		
INSPIRE support subscale	.30**	.31**	.15*	.66**	.73**	

^*^ indicates correlation is significant at the 0.05 level

^**^ indicates correlation is significant at the 0.01 level

**Fig. 1:**
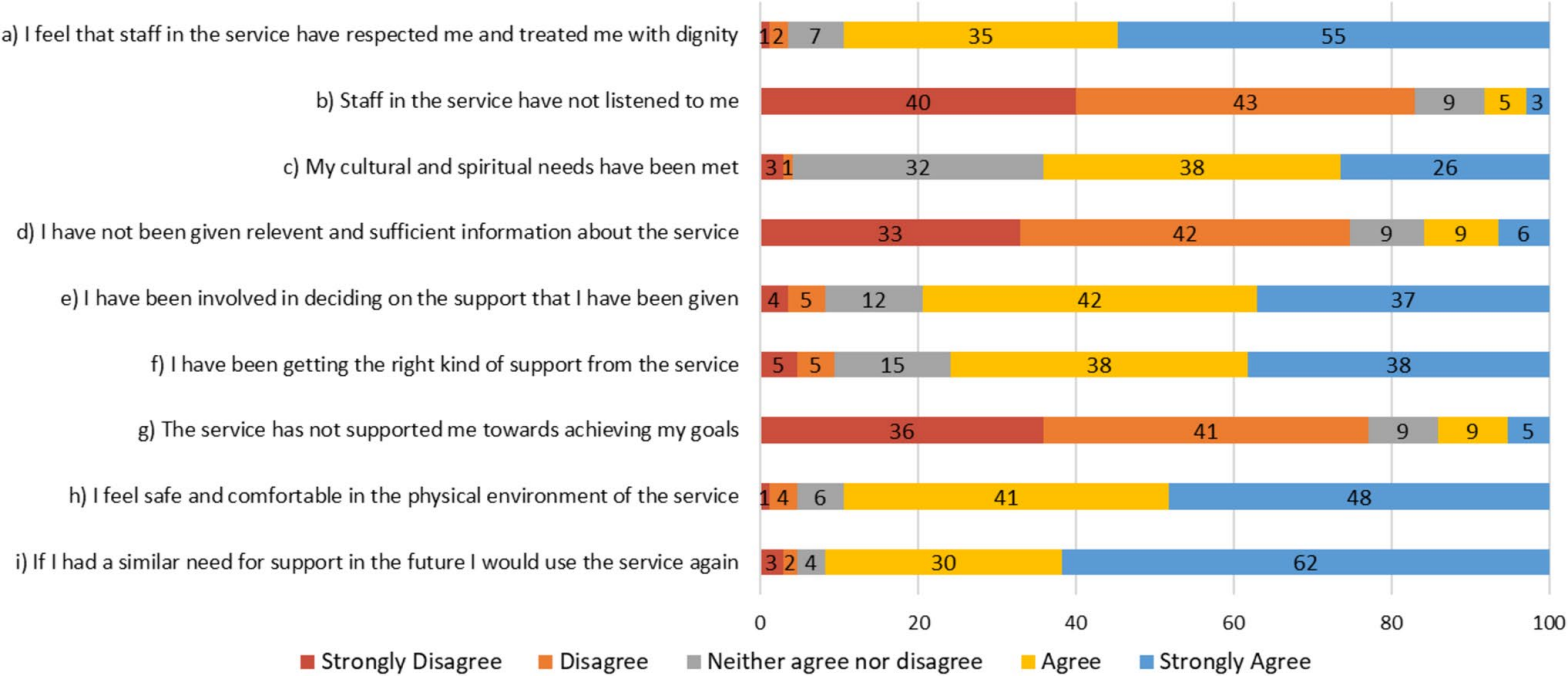
Ratings of MASS items (%)

Ratings for each MASS question provide insight into participants’ satisfaction with their PARC service stay (Fig. 1). The majority of responses were positive regarding all aspects of the PARC service stay (Fig. 1).

### Qualitative results

Four superordinate and 11 subordinate themes were identified from 170 responses to the three open-ended MASS questions. Of the 170 participants who responded to these questions, 166 answers were utilised in analysis as four participants did not respond to questions with enough detail to be analysed. Three superordinate themes came from participants’ views of the most helpful aspects of their PARC service stay: *Feeling Connected, Finding meaning and Purpose, and Self-Empowerment* were all embedded in participants’ recovery journeys. Participants’ views regarding what could be improved about PARC services were grouped under the superordinate theme of improvements to PARC services. These superordinate and subordinate themes, along with supporting quotes are presented in Table 4. Each of the four superordinate themes is also briefly described below.

**Table 4:** Superordinate and subordinate themes with quotes

Superordinate theme	Subordinate themes	Quotes
Feeling connected	(a) Support from staff	“The staff were very considerate, and they did listen and nothing seemed to be a bother for them. They always had time for you when you needed someone to listen and they were fantastic. I couldn’t have wished for better staff.”
		“…what they did was done without pressure and respecting your illness and waiting for you to recover in your own way…”
		“Having her [the peer worker] run the groups, it felt like she was speaking from experience. And her positive and endless energy was really supportive.”
	(b) Peer support and learning	“The most helpful thing was with being with other people that suffer from the same illness and being able to talk about it…and them being able to talk about themselves and how they cope with it. I am very isolated with my dad and it was good to have people around me—that’s what I needed.”
		“It was interesting meeting other people that have had a bit of a rough past, with mental illness as well. You’re not alone, you’re not alone dealing with this life thing and mental illness.”
	(c) Feeling part of a community and connections beyond PARC	“It’s just been meeting people that I’ve made friends with, and I have about 5 people I still have contact with.”
		“Just there being a constant supportive network there of people to talk to and work stuff out with other residents and staff.”
Finding meaning and purpose	(d) Participating in groups and outings	“They have helped me modulate my emotions through the sensory program. I’ve upskilled a great deal through that service. Some of the groups are psychosocial education and they have been really good.”
		“Just the supports in the groups, in all the groups and the way they conducted them. They were able to get things across to you in a way you could understand.”
		“Making new friends and going on day trips every day, e.g. to the movies, bowling. We went to the market as well a few times and a restaurant.”
	(e) Having daily responsibilities	“The group cooking was really helpful and everyone eating together, that was a big part of it and having responsibilities in the house. You were contributing, you had a role to play and responsible and you were part of the team.”
		“I got on very well with the staff and residents. They loved my cooking. We took turns every night, but I helped every night.”
Self-Empowerment	(f) Rebuilding myself	“Building my self-esteem, just giving me a bit of determination."
		“Confirming that I can succeed and giving me the confidence back that I had lost”
		“It was the first time I’d experienced hallucinations and fortnightly long flashbacks, so they actually helped me managed those. Learning how to manage those … has helped me managed them and feel less scared and I now feel in control and equipped to deal with them”
	(g) Having choice	“Being your own person. You still had you own freedom of taking your own medications, you’ve got to do it when you get home so, treating you as an adult so I liked that.”
		“You always had choice. They didn’t push you.”
		“The encouragement of independence—it was great that there was independence but there was also immediate support”
	(h) Being in a healthy and safe environment to recover	“Time to physically recover”
		“Helping me find peace”
		“Just being able to be in an environment in a safe environment, cos I was isolated for 12 months, being in a safe environment was really important…Just having a rest. Isolation can do a lot of damage when you’re unwell. Sometimes a safe place to sit is the best model, still having some interactions “The community meetings were important for us to have our say.”
Improvements to PARC services	(i) Increased support	“More workshops and one-on-one with staff.”
		“Only now I’m healing and that wasn’t talked about. More fostering the belief that you can recover because there was a lot of staff but they weren’t seeing people. They spent a lot of time in the office. Sometimes you wouldn’t see them all day.”
		“The meetings, the communication wasn’t right, and I felt like I didn’t have a clear cut plan of what was happening. I actually had to approach with one of the nurses about having a plan for leaving, like getting in touch with professionals, GP outside of the PARC, once I left.”
		“I felt like I was being forced out after my 2 week stay. I wasn’t ready to leave, and I think that is why I’m not doing so well”
		“They could also have more therapy. It would be good if they had a psychologist, more one-on-one contact. There was a lot of interaction in the first week and then it sort of dropped after that because they had to focus on new people”
	(j) Increased funding	“I just think they could have more of them. Because of waiting lists it can hard to get in when you need to. So if they had more PARC SERVICE you could get in more easily.”
	(k) Better facilities	“A lot of people are unaware that they can get better and this won’t be their whole life. Having books and knowledge that this isn’t the be and end all. Like having peers come in to talk about this, to show that they are dealing and it isn’t their whole life, having someone come in and talk about how they engage and where they go would be helpful. Having role models is really important.”
		“new mattresses… the beds are so old and uncomfortable which makes it hard to sleep.”
		“Having a pet as therapy, eg a cat.”

### Feeling connected

Feeling connected involved receiving and engaging with *support from staff*, engaging in *peer support and learning, and feelings of connection*.

Participants noted the importance of having someone there who would listen when they needed to talk, including peer and key workers, and often described this as 24/7 support [Table 4(a)]. Engaging in *peer support and learning* was identified as important to participants’ recovery journeys, including the opportunities to connect with others who have faced or are facing similar issues, and fostering a sense of not being alone through recovery [Table 4(b)]. Participants noted the PARC service environment also fostered these connections by facilitating new friendships, socialising, and helping to develop supportive networks [Table 4(c)]. The importance of feeling connected was emphasised by experiences when participants felt disconnected, further comments on the MASS mentioned social isolation and lack of social engagement prior to their PARC service stay.

### Finding meaning and purpose

Finding meaning and purpose involved *participating in groups and outings* and *having daily responsibilities.*

Finding meaning and purpose was viewed as important. Groups were described as important for participants in progressing their own recovery [Table 4(d)]. Participants described how *having daily responsibilities* and a sense of responsibility over the PARC service environment was helpful, consistent with the CHIME framework of recovery where finding meaning and purpose is crucial is one’s recovery journey. For example, one participant described how having responsibilities meant contributing to the upkeep of their residence and feeling part of a ‘*team*’ [Table 4(e)].

### Self-empowerment

Self-empowerment involved *rebuilding myself, having choice and being in a healthy and safe environment in which to start and continue one’s recovery*.

Self-empowerment included *rebuilding myself*, that related to regaining hope, confidence, self-esteem and new or old skills in oneself and one’s, life which one may gain through attending a PARC service. Some participants described the role of the staff in assisting them to gain skills to self-manage their mental health conditions and have choice in their recovery, including in regard to medications [Table 4(f)]. For some participants, *having choice in one’s life* was a key element in becoming empowered. Having choice over how they spent their time and being able to come and go as they pleased from the PARC service enabled participants to become more active, independent, and express choice in their lives during and beyond their PARC stay [Table 4(g)].

### Improvements to PARC services

Participants spoke about improvements to PARC services, which could be understood as interlinked; these included: *increased support, increased funding, and better facilities*.

The need for *increased support* was identified by some participants who reported either feeling unsupported in their recovery or not acknowledged by staff, with staff spending more time in the office rather than with consumers themselves; or feeling as if they were “forced out” of the service before they were ready. Suggestions for improving support included more supportive staff, increased interaction with staff, more extensive planning and support for discharge, more staff on shift, professional and communicative staff, and more groups and outings [Table 4(i)].

*Increased funding* for PARC services was a common suggestion to reduce waiting list times. Some participants described this as the need for greater accessibility and increased length of stay at PARC services [Table 4(j)]. Participants also suggested that PARC services could improve by having *better facilities* for engaging with peer support workers and other professionals, including one-on-one sessions with psychologists, as well as improving resources, such as better soundproofing and mattresses, a minibus for outings, internet access, and pets [Table 4(k)].

## Discussion

This study explored aspects of consumers’ self-reported personal recovery, in relation to their experiences and satisfaction after a stay at a subacute adult PARC service in Victoria, Australia. Generally, consumers reported improvements in their personal recovery and that the PARC service supported their recovery. Consumers also identified areas in which these services could be improved.

A significant increase in total QPR score at Time 2 suggests that a PARC service stay may contribute to short term personal recovery improvement. The overall positive increase in QPR scores indicate that PARC services have supported consumers to engage in the process of personal recovery. The significant change on the total QPR and intrapersonal subscale suggest that participants’ gains relating to self-empowerment and effective interpersonal relationships were improved or maintained in the weeks following their PARC service stays. These results are supported by our qualitative findings suggesting the benefits of connectedness with others during a PARC stay, albeit that significant change on the QPR interpersonal subscale scores was not detected. Our findings that intrapersonal and interpersonal QPR change scores were all moderately to strongly correlated are also consistent with previous studies using and testing the QPR [25, 31].

The INSPIRE validation study [26] and the REFOCUS Trial [5] in the UK have indicated average INSPIRE ratings of 72% for the Support sub-scale and 78% for the Relationships’ sub-scale amongst adults using community-mental health services primarily with a psychosis diagnosis. In Australia, the PULSAR trial [8] reported average INSPIRE ratings of 62% for the Support sub-scale and 75.5% for the Relationships sub-scale for people using adult community mental health services where staff had been trained in recovery oriented practice. In comparison, average INSPIRE ratings of recovery-oriented support among PARC service participants were similar to previous UK findings but higher than those reported in the PULSAR trial, whereas their average INSPIRE ratings of relationships with workers were higher than those reported in all three previous studies.

Satisfaction with services was also reflected in the MASS results. When examining individual items, consumers rated their experience of the PARC service positively on all nine items, with the vast majority stating that staff treated them with respect and dignity, and that they would use the service again if they required. This was further supported by the qualitative responses, with the PARC service stay assisting consumers to feel connected, find meaning and purpose, and become self-empowered. These three themes align with the CHIME framework, further indicating that PARC services foster personal recovery [28], and adds to the growing evidence that these services are largely well-regarded [21, 22, 32].

The significant correlations of the QPR with both the MASS and INSPIRE indicates that the process of recovery was associated with the quality of support provided by the PARC service, consistent with other literature on the meaning of ‘recovery’ in which the importance of support and quality of relationships with services are common themes [4, 33]. Authentic trauma-informed and recovery-oriented practice are shown to develop recovery narratives and support post-traumatic growth for individuals with experiences of mental health issues [4]. Furthermore, the significant link between satisfaction with services and improved personal recovery [21] indicates that implementing feedback collected from consumers may support PARC services in fulfilling their main objective to improve recovery outcomes for consumers. This further supports using personal recovery measures as routine measures in service evaluation to enable feedback for quality improvement. Based on the MASS results, the PARC services received quantitative and qualitative feedback indicating that they are successfully providing an environment that facilitates recovery, as well as supporting internal personal development. Similar themes have been identified by consumers in other studies, with activities, lifestyle change, therapeutic environment, and staff attributes highlighted as positive aspects of residential services [21, 22]. The most common suggestions for improvement related to the need for increased support, more peer involvement, and more effective communication of planning with consumers for leaving PARC services. Similar feedback has been provided in other studies, which have identified the need for increased goal setting and self-management [32]. Valuing consumers perspectives provides the opportunity for the aspirations of PARC services to be fully realised [34].

### Limitations and strengths

An important limitation is that this study did not include a control or comparison group, which means that any changes from Time 1 to Time 2 need to be interpreted cautiously and causal relationships can only be speculative. Also, following up with consumers within one week of their exit from the PARC service proved difficult due to difficulties obtaining information about exit dates and contacting participants. Therefore, many participants completed Time 2 measures more than 1 week after leaving the service, the mean (SD) days from exit to Time 2 data collection was 22.97 (34.86) [24]. As a result, recall bias may have impacted details reported about their PARC service stay for some participants. This issue was mitigated as much as possible by reminding participants to think back to their thoughts and feelings one week after their PARC stay. Additionally, one-third of the participants completed Time 1 measures only, so it is possible that people who did not complete the Time 2 measures were the most dissatisfied with the service, generating a bias towards positive feedback although many other factors may also influence decision making to participate in a study. The representativeness of the participants as a proportion of all PARC consumers during the 12-month study period is also unknown. Future research could account for this by gathering routine feedback measures during and upon exiting the PARC service to ensure diverse perspectives and experiences are included.

Despite the limitations, this study has contributed to the evidence base of the PARC services and has provided an insight into the links between satisfaction in service provision and recovery-oriented outcomes. A strength of this study is the relatively large number of participants across 19 of Victoria’s PARC services, enabling a cross section of perspectives. PARC services may implement this feedback to improve their capabilities for providing recovery-oriented care and to create avenues for further consumer input into service improvement. Additionally, whilst the involvement of lived experience researchers and valuing lived experience perspectives was crucial to this project, future research should involve lived experience researchers in higher levels of decision making; for example, at early stages of the research process, such as research design [35, 36]. This will contribute to developing a system that values and places consumers at the centre of mental health service delivery policy and practice reform.

## Conclusion

As part of one of the largest studies to investigate consumers’ personal recovery outcomes and satisfaction within sub-acute residential services, the findings highlight significant relationships between consumers’ satisfaction with service delivery and personal recovery outcomes. Consumer reported improvements across personal recovery domains after a PARC service stay, as measured by the QPR, suggest that the PARC services may support individuals’ personal recovery, consistent with the aim of these sub-acute residential services to provide recovery-oriented care. Further, the correlation between the services users satisfaction with the service and their own personal recovery indicates that it may be the supportive environment of the PARC service that contributes to engaging or re-gaging with a recovery narrative. Consumer satisfaction with services needs to be routinely evaluated and response to feedback needs to be actioned to achieve the best possible recovery-oriented services, outcomes for consumers and to continue to build evidence-informed sub-acute community-based residential services.

## Data Availability

The data that support the findings of this study are not openly available due to reasons of sensitivity and are available from the corresponding author upon reasonable request.
